# A Review on Fully Bio-Based Materials Development from Polylactide and Cellulose Nanowhiskers

**DOI:** 10.3390/polym14194009

**Published:** 2022-09-25

**Authors:** Purba Purnama, Muhammad Samsuri, Ihsan Iswaldi

**Affiliations:** 1 School of Applied STEM, Universitas Prasetiya Mulya, Tangerang 15339, Banten, Indonesia; 2 Vanadia Utama Science and Technology, PT Vanadia Utama, Jakarta 14470, Indonesia; 3 Chemical Engineering Department, Universitas Bhayangkara Jakarta Raya, Bekasi 17121, West Java, Indonesia

**Keywords:** polylactide, cellulose nanowhiskers, biopolymer, nanocomposite, stereocomplex, interfacial compatibility, nucleating agent

## Abstract

This review covers the development of eco-friendly, bio-based materials based on polylactide (PLA) and cellulose nanowhiskers (CNWs). As a biodegradable polymer, PLA is one of the promising materials to replace petroleum-based polymers. In the field of nanocomposites, CNWs offer many advantages; they are made from renewable resources and exhibit beneficial mechanical and thermal properties in combination with polymer matrix. A wide range of surface modifications has been done to improve the miscibility of CNW with the PLA homopolymer, which generally gives rise to hydrophobic properties. PLA–CNW nanocomposite materials are fully degradable and sustainable and also offer improved mechanical and thermal properties. Limitations pertaining to the miscibility of CNWs with PLA were solved through surface modification and chemical grafting on the CNW surfaces. Further development has been done by combining PLA-based material via stereocomplexation approaches in the presence of CNW particles, known as bio-stereo-nanocomposite PLA–CNW. The combination of stereocomplex crystalline structures in the presence of well-distributed CNW particles produces synergetic effects that enhance the mechanical and thermal properties, including stereocomplex memory (melt stability). The bio-based materials from PLA and CNWs may serve as eco-friendly materials owing to their sustainability (obtained from renewable resources), biodegradability, and tunability properties.

## 1. Introduction

Petroleum-based and bio-based polymers are widely used for various purposes. Petroleum-based polymers, including polyethylene (PE), polypropylene (PP), polystyrene (PS), high-impact polystyrene (HIPS), polyvinyl chloride (PVC)polyethylene terephthalate (PET), polycarbonate (PC); are widely used in the packaging, electronics, and automotive industries, among others. Petroleum-based polymers are cheaper than bio-based polymers but are non-degradable. The high consumption of petroleum-based polymers in various fields causes tremendous environmental issues including air and soil pollution. Incineration of non-degradable polymers always produces large amounts of carbon dioxide, thereby contributing to global warming; moreover, it sometimes produces toxic gases that also contribute to air pollution worldwide. Thus, recycling petroleum-based polymers may help protect the environment. Petroleum sources are finite, non-renewable, non-sustainable, and on the brink of exhaustion.

In contrast, bio-based polymers are defined as polymeric materials derived from renewable resources. Biodegradable polymers are polymers that can fully degrade by microorganisms through physical and chemical deterioration processes [1]. Bio-based polymers are not the same as biodegradable polymers, which means that not all bio-based polymers are biodegradable and vice versa. Therefore, in this review bio-based polymers refer to bio-based polymers with biodegradable properties.

Because bio-based polymers are biodegradable and made from renewable resources, they have the potential to reduce environmental problems and preserve sustainability. However, bio-based polymers have certain limitations in their physical, thermal, and mechanical properties as compared with petroleum-based polymers [2]. Consequently, the properties of bio-based polymers should be modified before their use as a replacement for conventional polymers in various applications.

Environmental legislation drives the development of various biodegradable polymers as an alternative to petroleum-based polymers, including polylactide (PLA), polyglycolic acid (PGA), polycaprolactone (PCL), polyhydroxyalkanoates (PHAs), starches, poly(butylene succinate) (PBS), and polyhydroxy butyrates (PHBs). Of these, PLA is a bio-based polymer that offers outstanding properties. It is a well-known “eco-friendly polymer” produced from biodegradable agricultural resources [3,4]. It has remarkable properties, including biodegradability, biocompatibility, UV stability, and processability; moreover, it has great potential in various advanced applications such as the development of high-performance material for use in the automotive industry and biomedical applications. However, PLA has some limitations; it requires certain modifications to overcome its thermal and mechanical property-related shortcomings. PLA can be combined with other materials to achieve acceptable properties. Many researchers have reported the development of PLA-based materials through the addition of other polymers [5,6,7], stereocomplexation [8,9,10,11,12,13,14], and through the addition of inorganic materials as reinforcing fillers [15,16,17,18]. The morphology, thermal behavior, interfacial, and rheological properties were studied from PBS/PLA and PCL/PLA blends, which strongly depend on polymer miscibility, were studied [5,6]. Interfacial adhesion and the morphology of the polymer blends were responsible for the improvements in their properties [7]. Stereocomplexation led to the improved thermal and mechanical properties of PLA-based materials via the formation of stereocomplex crystalline structures [8,9]. Functional hybrid materials offer a synergetic assembly of biopolymers and nano-sized inorganic particles [15]. With a combination of organic polymers with nano-sized inorganic materials, polymer nanocomposites showed significant improvements in their thermal, mechanical, and barrier properties [17]. Nano-sized cellulose particles with good interfacial interactions with polymer matrix are responsible for the enhancement of the properties of polymer nanocomposites [18]. Nature provides an unlimited source of plant- or animal-derived biomaterials, including biopolymers and bio-based nanoparticles. To avoid environmental damage, the use of degradable fillers derived from renewable resources such as cellulose-based fillers is preferred. The use of cellulose nanowhiskers (CNWs) attracted great interest owing to their advantages, including their processability, recyclability, high aspect ratio, high strength and stiffness, degradability, and renewable characteristics [18,19,20,21,22].

In the future, the development of biomaterials should focus on sustainability, degradability, and the tunability of properties. Of all the bio-based materials, PLA and CNW comply with the future materials development requirements; moreover, these materials have specific characteristics. PLA can be developed as an advanced material through nanocomposites [22,23,24,25,26,27,28] and stereocomplexation [29,30]. Furthermore, the combination of PLA-based materials and CNWs offers the synergetic properties enhancement of these materials [18,22,29,30], preserving their renewable and degradable characteristics. The molecular structure adaptability of PLA and CNW through molecular and structure modifications helps overcome the main problems concerning their miscibility. Herein, we reviewed the development of fully bio-based materials derived from PLA and CNW blends and assessed the over process and property enhancements. We first present a discussion of some general aspects of PLA and CNWs. The synergetic effects of the PLA and CNW blend on the improvement of the properties of fully bio-based PLA and CNW materials are also discussed.

## 2. Synthesis and Properties of PLA and CNWs

### 2.1. Polylactide (PLA)

PLA is an important aliphatic polyester comprising repeating units of lactide. It is considered a “green polymer” because it is produced from renewable resources. Lactide is a cyclic dimer prepared from lactic acid dehydration through a controlled depolymerization process. Lactic acid is a chiral molecule and exists as L and D isomers. It can be obtained through the fermentation of corn, sugar cane, sugar beet, etc [31,32]. Lactide has three different stereoisomers: L-lactide, D-lactide and meso-lactide (D,L-lactide); thus, the ring-opening polymerization of lactide produces poly(L-lactide) (PLLA), poly(D-lactide) (PDLA) and poly(D,L-lactide) (PDLLA), respectively.

**Figure 1:**
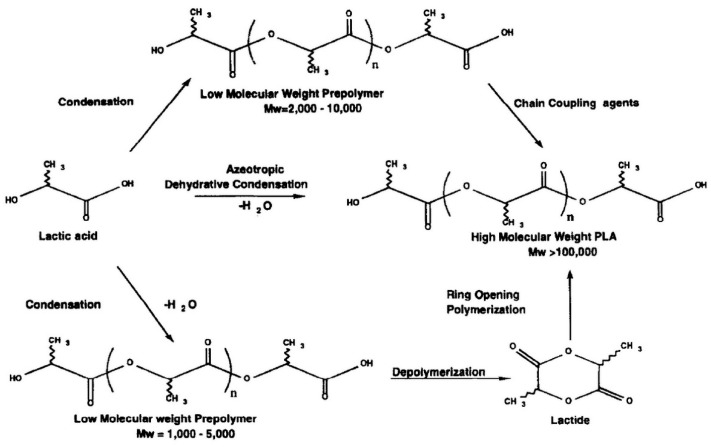
Polymerization route of polylactide. (Copyright and permission, [32]).

PLA is formed either through the direct polycondensation of lactic acid monomers or by cyclic intermediate dimers (lactide) followed by the ring-opening polymerization process, as shown in Figure 1 [32]. In the polycondensation process, water is removed through condensation and the use of a solvent under high vacuum and temperature conditions. This process produces low-to-intermediate molecular weight PLA owing to the difficulties associated with the removal of water and its impurities. However, this process has some disadvantages: it requires a large reactor, an evaporation step, and solvent recovery, and has a high chance of racemization. Mitsui Toatsu used a solvent-based process to produce high-molecular-weight PLA through direct condensation with azeotropic distillation to continuously remove water from the condensation process [33].

Ring-opening polymerization is superior to better than polycondensation in that it produces high-molecular-weight PLA. This process removes water using mild conditions and without the need for an organic solvent, thereby producing a lactide intermediate that can be subjected to vacuum distillation for purification. NatureWorks LLC has developed and patented a low-cost continuous process for the production of PLA with a controlled molecular weight [34]. The molecular weight range can be predicted by controlling the lactide dimers purity and catalyst ratio. In the ring-opening polymerization process, many catalysts can be used for polymerization, including stannous octoate [35,36,37,38,39], Al-alkoxides [40,41], yttrium and lanthanide alkoxides [42], and iron alkoxides [43]. Stannous octoate is a common catalyst used to produce high-molecular-weight PLA with stereoregularity through a coordination–insertion mechanism [36]. Yttrium and lanthanide alkoxide support good polymerization activity [42]. Aluminum alkoxide can be used to produce high-molecular-weight PLA with a homogenous chain length or narrow molecular-weight distribution [41]. Generally, ring-opening polymerization can be performed under heat [34,35], the presence of solvent [33,37,40,41], or supercritical fluid conditions [38].

PLA is a potential replacement for petroleum-based polymers (PE, PET, PS, PC) owing to its biodegradability and biocompatibility properties [32,44] and is being produced on a large scale since 2001 [3]. PLA is a thermoplastic polymer and has properties similar to those of PS and PET as shown in Table 1 [45,46,47]. At room temperature, PLA is a stiff and brittle biopolymer with a glass transition temperature of approximately 55 °C and a melting temperature of 180 °C, depending on its crystallinity and molecular structures. Isotactic PLLA and PDLA are mostly high crystalline polymers, whereas PDLLA is an amorphous material [48]. Although PLA-based materials with high crystallinity have good physical, mechanical, and thermal properties, including biocompatibility, they have a slow degradation process. PLA is now being used as an alternative to petroleum-based polymers such as PET, PVC, HIPS in the packaging industry. PLA can also be combined with other materials for use in specific applications.

### 2.2. Cellulose Nano-Whiskers (CNW)

Cellulose is a bio-based material derived from renewable resources, such as plants, bacteria, trees, and tunicate, and is synthesized from glucose through condensation polymerization. During this process, a long chain of anhydro-glucose units joins together through the formation of β-1,4-glycosidic bonds [49]. Cellulose generally exists in the form of cellulose fiber, microcrystalline cellulose, and nanosized cellulose [49,50]. Of all these, as nanosized cellulose crystals, CNWs are attracting great interest, as evidenced by the increasing number of publications over the past decades [18,19,20,21,22,23,24,25,26,27,28,29,30,51,52,53,54,55,56,57,58,59,60,61,62].

**Figure 2:**
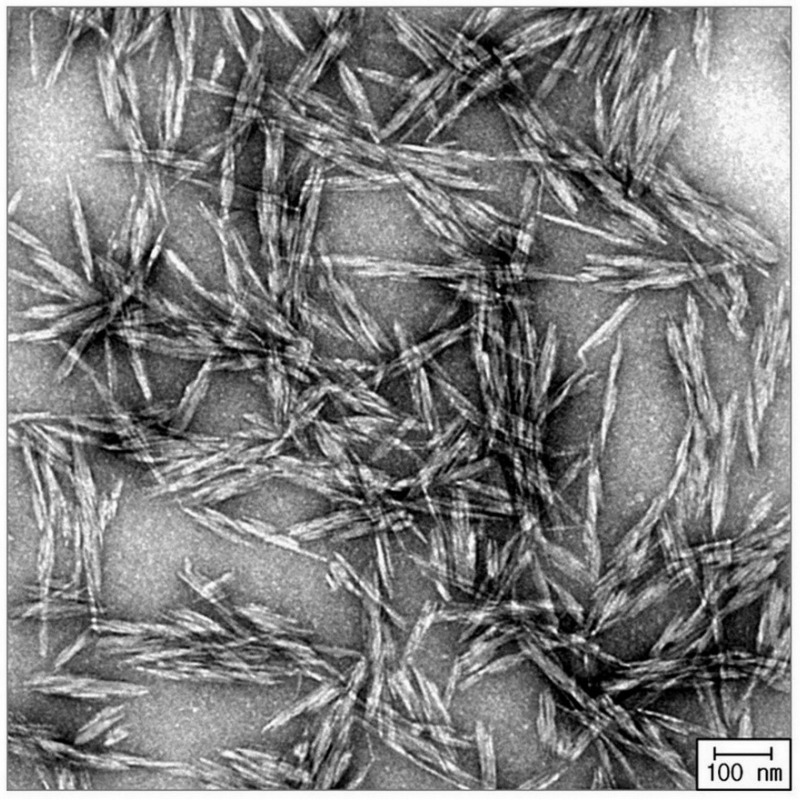
Transmission Electron Microscopy (TEM) image of acetylated CNW isolated from hydrolysis microcrystalline cellulose. (Copyright and permission, [29]).

Cellulose sources are not completely crystalline but are a combination of crystalline and amorphous. The amorphous nature renders the cellulose structure sensitive to acid hydrolysis followed by breakdown into single crystallites. CNWs can be produced from various cellulosic materials through acid hydrolysis [18,51,52,53]. The dimensions of the CNWs depend on the initial source and hydrolysis conditions [54]. For some cellulosic materials, different hydrolysis times result in different dimensions of the CNWs [55,56]. The dimensions of the CNWs obtained from the acid hydrolysis of cellulose range from 200 to 500 nm in length and from 3 to 30 nm in diameter [57]. Rod-like CNWs can be generated by controlling the concentrations of the cellulosic materials and sulfuric acid, temperature, and ultrasonic treatment time [55]. The success of CNW synthesis can be confirmed using transmission electron microscopy (TEM) [18,29,53], atomic force microscopy (AFM) [30], and functional group analysis using Fourier transform infrared (FTIR) spectroscopy [18,58]. Typical structures of some CNWs obtained from the acid hydrolysis of cellulose are shown in Figure 2 [29].

CNWs are generally semicrystalline, and their properties depend on their source. Using X-ray diffraction, Sakurada et al. determined that the crystal modulus of CNWs from cellulose-I is 138 GPa; a similar value was noted for CNWs from plant cellulose fibers [60]. Sturcova et al. isolated CNWs from tunicin with a tensile modulus of approximately 143 GPa [61], whereas Rusli and Eichhorn isolated CNWs from cotton with a tensile modulus of approximately 57–105 GPa [62]. Determining the mechanical properties of very small particles is really difficult. By theoretical calculation, modulus values ranging from 100–160 GPa can be obtained through molecular dynamics/mechanics methods [63]. These modulus values are indicative that the particles may be used as fillers or additives.

### 2.3. PLA and CNW Compatibility

The main things to consider for future materials include supply continuity (use of renewable resources), properties (physical and mechanical), and processability. PLA and CNWs are potential bioresources with future prospects. As biodegradable and biocompatible material, PLA has some shortcomings in its mechanical and thermal properties. CNWs are interesting materials owing to their excellent mechanical and thermal properties; moreover, they are the most abundant materials on the planet and are renewable, inexpensive, and easy to process. Combining PLA and CNWs helps enhance the individual materials’ properties without losing their biodegradability [64]. Material compatibility is the key to ensuring the formation of homogenous blends with other materials and is strongly dependent on surface functionality. As derivatives of polysaccharides, CNWs are not miscible with hydrophobic polymers [65]. When hydrophilic functional groups are present on CNWs surfaces, the interfacial compatibility of PLA with CNWs is limited. Consequently, hydrophilic CNWs exhibit weak interfacial bonding and dispersion in the PLA matrix, which affects their thermal and mechanical properties [66].

The dispersion of the CNWs in the solvent or polymer matrix is also affected by the type of acid used in the hydrolysis process [67]. When sulfuric acid hydrolysis is used, CNWs have better dispersion in aqueous solvents, owing to electrostatic repulsion from the negatively charged (anionic) sulfonate ester groups [55]. CNWs are highly reactive and functionalizable, owing to their high surface-to-volume ratios, and contain many surface hydroxyl groups (–OH) [67]. Moreover, surface modifications are required to improve the CNW-polymer interface in polymer nanocomposite systems. Substitution of the surface hydroxyl groups on the CNW surfaces with organic molecules and polymer grafting can help improve the hydrophobic ability of the CNWs or their interfacial compatibility with the PLA matrix [66].

**Figure 3:**
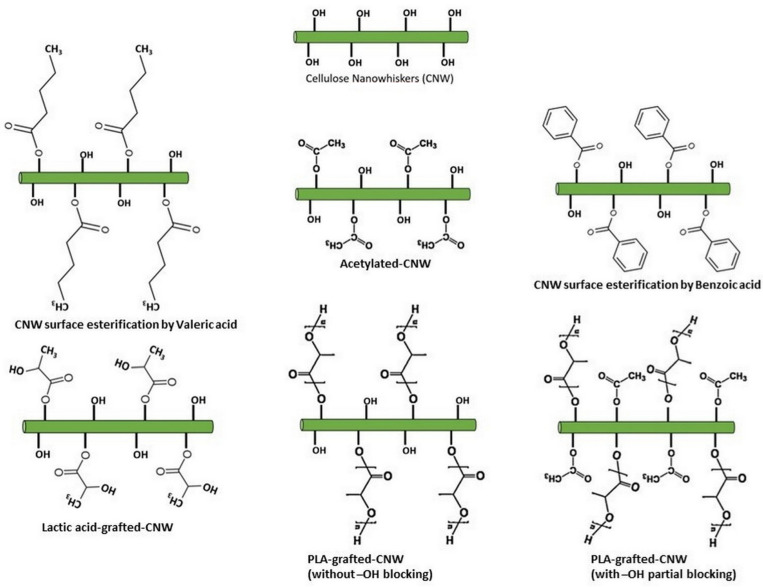
Illustration of surface modifications of CNW particles.

Many studies have utilized certain special chemicals to improve CNW dispersion in organic solvents, including poly(ethylene glycol) (PEG) [63], silanes [68], n-octadecyl isocyanate [69], hydrochloric acid combine with acetic acid [18,29,30,70,71], sulfuric acid [58], and polyvinyl alcohol [72]. Some surface modifications have been performed to improve the miscibility of CNW with the PLA matrix, as illustrated in Figure 3. Moreover, the –OH functional group can be substituted with other hydrophobic molecules through acetylation [18,29,30], esterification with organic acid (benzoic acid, valeric acid) [73,74], and lactic acid [75]. Substitution of these –OH functional groups with hydrophobic chains diminishes the hydrophilicity of the CNWs surface and increases its hydrophobicity based on the replacement portion. These strategies improve the dispersion of CNWs in the PLA matrix. PLA molecules can also be grafted on the CNW surface by controlling the number of –OH functional groups as a co-initiator during polymerization [28,58]. Unmodified CNWs containing many –OH functional groups result in low-molecular-weight grafted-PLA chains. Blocking the surface hydroxyl groups of CNWs support the ring-opening polymerization of lactide to obtain high-molecular-weight PLA [18,29,30,58]. The surface modifications of CNWs improve their dispersibility in the PLA matrix and preserves their high mechanical properties, crystallinity, and degradability [76].

## 3. PLA–CNW Nanocomposites

Nanocomposites reportedly improve the mechanical and physical properties of the polymer matrix [15,16,17]. Among all the available nanoparticles, CNWs are natural organic-based nanoparticles and are considered promising nanomaterials owing to their cheap price and abundance in nature. PLA and CNW blends may replace petroleum-based polymers and provide advantages such as property tunability, degradability, and resource sustainability.

Some studies have demonstrated the direct utilization of cellulose-based materials to improve the properties of PLA [70,77,78,79]. The biocomposite PLA and natural fibers almost achieved the mechanical properties of glass fiber-reinforced polymers [77]. Oksman et al. reported on a promising composite of PLA and flax fibers with a significant improvement in stiffness at 30% of the flax fiber content [78]. The excess –OH groups on the cellulose fiber surface increase its reactive compatibility with the PLA matrix but restrict the molecular weight of PLA [79]. The portion of the substitution of microfibrillated cellulose –OH groups with acetyl functional groups affects the mechanical properties of PLA-microfibrillated cellulose composites [70]. Hydrophilic surfaces limit cellulose dispersion in the PLA matrix. Furthermore, a high cellulose particle content affects the optical properties of the blended materials. It is thus important to control the surface hydrophilicity and minimize the particle loading of cellulose-based materials.

CNWs are nano-sized and whisker-like crystalline particles isolated from cellulose through the process of acid hydrolysis. Nano-sized CNWs with excellent dispersion in polymer matrix results in impressive improvement in the mechanical properties at low loading levels through a percolation mechanism supported by hydrogen bonds [80].

Many researchers have studied the combination of PLA matrix with CNWs (neat and modified surfaces) to improve their physical, mechanical, barrier-related, and thermal properties, as presented in Table 2. The addition of CNWs with abundant surface hydroxyl groups from the acid hydrolysis of cellulose enhanced the mechanical, thermal, and barrier-related properties of hydrophobic PLA matrix with a high molecular weight (*M*_w_ > 100,000 gr/mol) through solution casting [22,26,27]. This enhancement of properties was attributed to the char formation and increased crystallinity [22].

**Figure 4:**
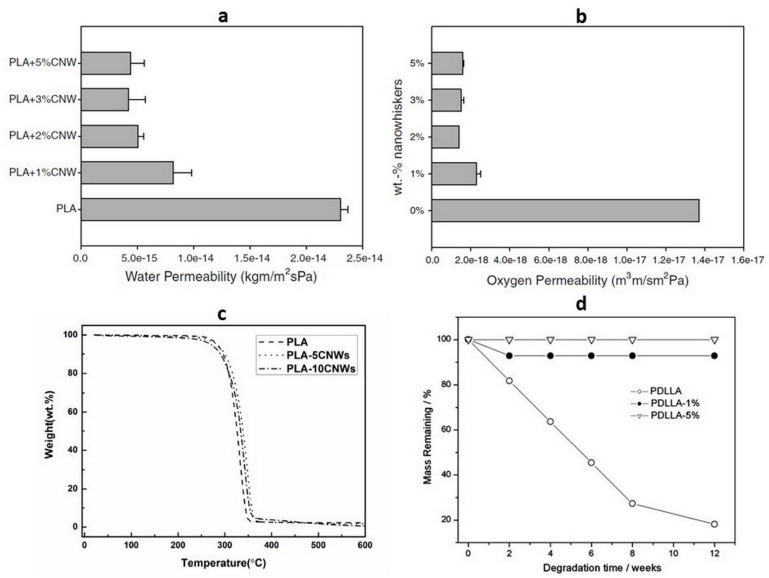
(**a**). Water permeability of PLA and their nanocomposites with various CNW content.; (**b**). Oxygen permeability of PLA and their nanocomposites with various CNW content. (Copyright and permission, [26]); (**c**). Thermogravimetric analysis (TGA) thermogram of the PLA and PLA–CNW; (**d**). Residual mass of the neat PDLLA and its nanocomposites as a function of degradation time. (Copyright and permission, [83]).

Sanchez-Garcia and Lagaron used CNWs as a reinforcing agent in the polylactide matrix to reduce the gas and vapor permeability: optimum barrier enhancement was obtained with CNWs at low loadings, as shown in Figure 4a,b [26]. The addition of 3% well-distributed CNW particles in the PLA matrix reduced the water and oxygen permeability by approximately 82% and 90%, respectively. The presence of crystalline CNW materials affects the barrier properties of the PLA–CNW materials due to filler-induced nucleation and results in increased crystallinity [26], whereas the surface hydroxyl groups improve the water absorption of the PLA nanocomposites [27]. A degradation study was performed; compare with the original PLA homopolymers, low-loading unmodified CNWs showed better stability at a temperature of 310 °C (Figure 4c) [27]. The presence of CNWs without surface modifications delays the hydrolytic degradation of the PDLLA matrix (Figure 4d) because these CNWs function as a physical barrier and prevent water diffusion to polymer molecules, thereby re-routing the kinetics of hydrolytic degradation [83].

The surface hydroxyl group on CNWs limits their compatibility and dispersion in the PLA matrix. Controlling the number of the –OH groups on the CNWs helps increase the miscibility of the CNWs with PLA matrix and control their hydrophilicity. Therefore, CNW surface modifications are required to achieve acceptable dispersion and compatibility levels of CNWs in the PLA matrix. The polarity of CNWs can be decreased by the blocking effect of the acetyl group on the CNW surface, leading to enhanced CNW dispersion in PLA and accordingly improving the physical and mechanical properties of the PLA–CNW nanocomposites [18,25]. The interaction between the acetyl groups on the CNW surface and PLA was evidenced by the shift in the carbonyl stretching spectra at 1748 cm^−1^ [84]. Other organic CNW surface modifiers mainly focus on reducing the surface hydroxyl groups through esterification to successfully increase the CNW dispersion in the PLA matrix [73,74,83,84,85,86]. The shielding effect of triazine derivatives on the CNW surface supports the improvement of the thermal degradation properties of the PLA–CNW nanocomposites [23,86]. Well-dispersed silanized CNWs in the PLA matrix are a result of the strong hydrogen bonding of –COOH groups in PLA and the –OH groups in the silanized CNWs [87]. The functionalization of the CNW surface was also enhanced by the addition of a radical initiator dicumyl peroxide through grafting between the PLA methine (–CH) groups with methylene groups of the CNWs [88].

Compared with the functional groups, the grafted-PLA chains on the CNWs reportedly increases the miscibility. It can be obtained via in situ ring-opening polymerization [18,28,58,90,91]. Because –OH groups on the CNW surface can be controlled by the partial blocking of organic molecules, these –OH groups can be used as co-initiators to graft a PLA chain onto the CNW surface. The in-situ polymerization of lactide onto unmodified CNWs results in low-molecular-weight PLA chains owing to the excess –OH groups present on the CNW particles [58]. Partially-blocked –OH groups on the CNW surface led to effective ring-opening polymerization of lactide, resulting in the formation of high-molecular-weight PLA chains [18]. High-molecular-weight PLA–graft–CNW tackles the hydrophobic/hydrophilic incompatibilities of the PLA–CNW nanocomposites and consequently, leads to tremendous improvements in the material properties: thermal stability, crystallinity, mechanical, barrier-related, optical, and morphological properties [28,58,90,91]. The PLA–graft–CNW improves the miscibility of the CNWs with PLA matrix, resulting in the homogenous dispersion of the CNW particles. Dispersed CNW particles accelerate the formation of nucleating sites and enhance the crystallization ability of PLA matrix [58]. PLA–CNW nanocomposites can be easily obtained in the presence of organic solvents [18,22,25,26,27,75,81,82,83,84,85,87,90] or be produced using thermal mixing process [23,28,58,73,74,86,88,89], which also suitable for high-molecular-weight PLA matrix [25,74,90].

## 4. Bio-Stereo-Nanocomposites PLA–CNW

**Figure 5:**
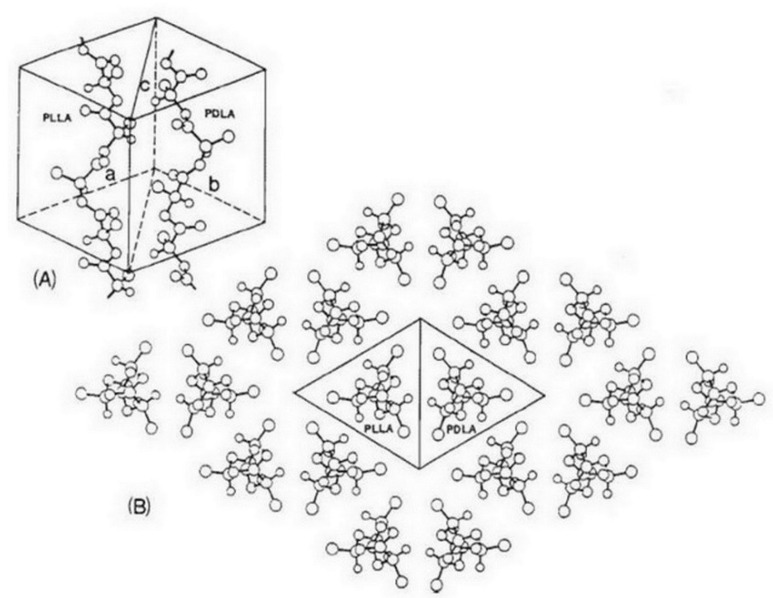
Crystal structure of s-PLA. (**A**) Structural model of the stereocomplex of PLLA and PDLA with the space group p l. In the present case, the pointing direction of methyl groups is upward. (**B**) Molecular arrangement projected on the plane normal to the chain axis. (Copyright and permission, [9]).

Nanocomposite is not the only method that can be used to improve the thermal and mechanical properties of PLA. Stereocomplex PLA (s-PLA) is another method to improve the properties of PLA. S-PLA is an enantiomeric polymer blend of PLLA and PDLA [8]. The crystal structure of s-PLA is different from that of its homopolymer as shown in wide-angle x-ray spectroscopy (WAXS) profiles. The main peaks of the PLA homopolymer (*X_D_* = 1) appeared at 2*θ* values of 15°, 17°, and 19°, whereas the main peaks of s-PLA (*X_D_* = 0.5) were observed at 2*θ* values of 12°, 21°, and 24° [8]. Okihara et al. proposed the crystal structure of s-PLA (Figure 5) [9]. The thermal and mechanical properties of s-PLA improved because s-PLA is strongly bounded by the crystal network and exhibits decreased motion as compared with PLA homopolymers.

S-PLA can be produced using the solution [92,93,94], melt blending [95,96,97], supercritical fluid [13,14,98], and microwave irradiation method [99], which is mostly similar to the synthesis route of PLA–CNW nanocomposites. Therefore, both stereocomplexation and nanocomposite approaches can be applied using the same method for the combination of PLA and CNW materials. The molecular weight of PLA (critical value: approximately 100,000 g/mol) is an additional hindrance to the formation of stereocomplex crystallites [100].

Bio-stereo-nanocomposites are defined as advanced materials developed from biodegradable materials by combining stereocomplexation and nanocomposite approaches. The bio-stereo-nanocomposite PLA–CNW is the combination of fully bio-based PLA and CNW through stereocomplexation and nanocomposite approaches. As an advanced biomaterial, bio-stereo-nanocomposite PLA–CNW offers many benefits for wider application in the future: biodegradability, sustainability, and tune-ability of the thermal-mechanical properties. Bio-stereo-nanocomposite PLA–CNW can be synthesized by combining enantiomeric PLA mixtures with pristine or surface-modified CNWs or by mixing graft structures such as CNW-grafted PDLA and PLLA.

**Figure 6:**
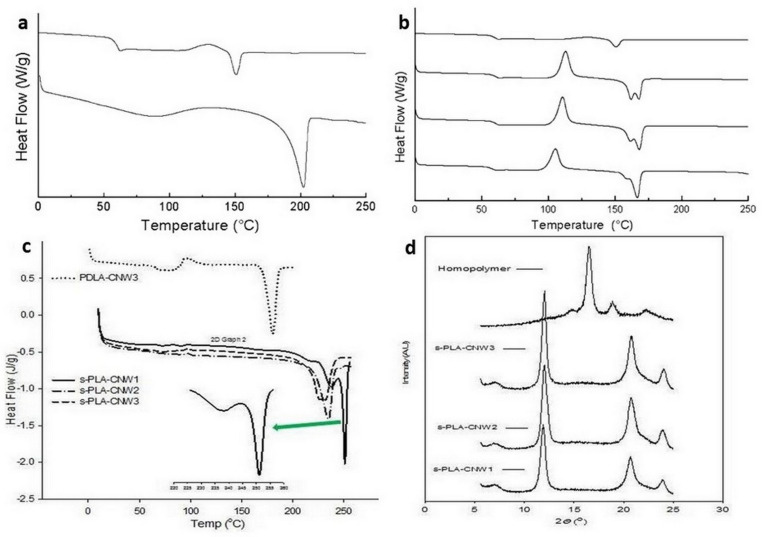
(**a**). DSC thermogram of neat PLLA (**top**) and PLLA/CNW-grafted PDLA at 50:50 ratio; (**b**). DSC thermogram of neat PLLA (**top**) and PLLA/CNW-grafted PDLA at different content 2.5, 5, and 10% (**top** to **bottom**).; (**c**). DSC thermogram of the PDLA-CNW3 (homopolymer) and generated bio-stereo-nanocomposite PLA–CNW materials; (**d**). XRD diffractogram of homopolymer PLA–CNW and generated bio-stereo-nanocomposite PLA–CNW materials. (Copyright and permission, [29]).

Bio-stereo-nanocomposite PLA–CNW was first developed through solution casting of PLLA and CNW-grafted PDLA at a 50:50 weight ratio and was found to contain excellent stereocomplex crystallites [101]. The addition of CNW-grafted PDLA at a low amount (up to 10% weight ratio) also supports the formation of stereocomplex crystallites concurrently with PLA homocrystallites [102,103]. The formation of stereocomplex crystallites was confirmed using differential scanning calorimeter (DSC) analysis, the results of which are shown in Figure 6a,b [102]. A single melting temperature (*T*_m_) of 220 °C for the equimolar ratio indicated the presence of stereocomplex crystallites, whereas a lower CNW-grafted PDLA indicated the presence of homocrystallites [102]. Cao et.al. produced a bio-stereo-nanocomposite PLA–CNW with partial stereocomplex crystallites from a mixture of PLLA and PDLA containing 5% acetylated CNW particles [104]. However, the generated bio-stereo-nanocomposite did not adequately support the formation of s-PLA crystallites from high-molecular-weight PLLA and PDLA blends because the CNW particles could not prevail in the formation of homocrystallites during the crystallization process for high molecular weight (Mw > 100,000). The bio-stereo-nanocomposites PLA–CNW was also successfully developed by mixing CNW-grafted PLLA and PDLA at a 50:50 weight ratio using solution casting [30] and supercritical carbon dioxide–dichloromethane [29]. The formation of a perfect stereocomplex in the bio-stereo-nanocomposite PLA–CNW was confirmed using the DSC and X-ray diffraction (XRD) analyses, as shown in Figure 6c,d [29]. The DSC thermograms of the bio-stereo-nanocomposite PLA–CNW with various CNW loadings (s-PLAC-CNW1, s-PLA–CNW2, and s-PLA–CNW3) exhibited a single *T*_m_ of approximately ~230 °C without the homopolymer *T*_m_ (approximately ~180 °C). Perfect stereocomplexation was also confirmed through characteristic diffraction peaks of s-PLA at 11.86°, 20.56°, and 23.86° of 2*θ* value (Figure 6d).

Bio-stereo-nanocomposites PLA–CNW from the CNW-grafted PLLA and PDLA showed unique processing characteristics [29]. The formation of stereocomplex crystallites in the solution is strongly related to the solubility of the polymer matrix in the solvent. The graft structure of PLA–CNW nanocomposites magnifies the number of functional groups that reduce the polymer viscosity in organic solvents. Contrary to the formation of linear s-PLA, unusual stereocomplex crystallite formation was found for bio-stereo-nanocomposites PLA–CNW. The well-distributed CNW particles and the reducing solution viscosity resulted in a higher possibility of grafted-PLLA and grafted-PDLA chains interacting and immediately forming stereocomplex crystallites. Bio-stereo-nanocomposites PLA–CNW can be obtained using the solution method in a relatively short time (approximately 5 min) from various molecular weights (low, medium, and high) and concentrations of CNW-grafted PLLA and PDLA [29].

**Figure 7:**
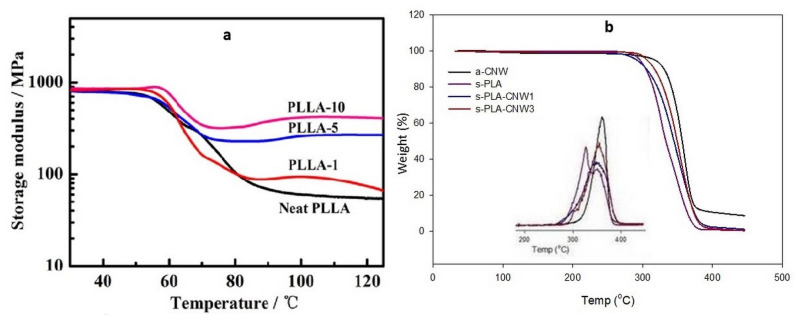
(**a**). Storage modulus 9E’—temperature curve of neat PLLA and CNW-grafted-PLLA at different content (PLLA-1; PLLA-5; and PLLA-10) [103]; (**b**). TGA traces of a-CNW, s-PLA, and bio-stereo-nanocomposite with different CNW contents (s-PLA–CNW1, and s-PLA–CNW3) under nitrogen atmosphere. The insert shows the first derivative of the weight loss. (Copyright and permission, [30]).

Neat s-PLA materials have improved mechanical and thermal properties compare with PLA homopolymers. The addition of unmodified or surface-modified CNW particles in the stereocomplex matrix can enhance its properties to a greater extent. As mentioned in a previous report, s-PLA crystallites increase the tensile strength and Young’s modulus up to 25% more than neat homopolymers do [13]. In a study focusing on polymer nanocomposites, well-distributed nanoparticles also improved the mechanical properties of polymeric materials [105]. Bio-stereo-nanocomposite PLA–CNW showed enhanced mechanical and thermal properties despite the low content of the CNW particles. The presence of CNW particles (3% content) as a core structure in bio-stereo-nanocomposites PLA–CNW (s-PLA–CNW3) enhances its mechanical properties more than that in neat s-PLA materials (up to 2.70 GPa and 62.96 MPa for Young’s modulus and tensile strength, respectively) [30]. Moreover, the addition of 10% CNW-grafted PDLA in the PLLA matrix also improves the storage modulus E’ at approximately 4.15 GPa (at 25 °C) and 0.66 GPa (at 100 °C) as compared to neat PLLA (approximately 2.93 GPa and 0.41 GPa at 25 °C and 100 °C, respectively) [102]. These property enhancements are most probably caused by the reinforcing effect of CNW crystallites through hydrogen bonding and the stabilizing percolation network by stereocomplex crystallization [102]. Wu et al. reported that the addition of 10% CNW-grafted-PDLA in the PLLA matrix shows significant s-PLA crystallites which act as nucleation sites for PLLA homopolymers; accordingly, notable improvement was observed in the storage modulus at high temperatures as shown in Figure 7a [103].

Bio-stereo-nanocomposite PLA–CNW also shows significant enhancements in its thermal properties. A comparison of acetylated CNW, neat s-PLA, and bio-stereo-nanocomposite PLA–CNW (s-PLA–CNW) showed that the presence of CNW particles improves the thermal decomposition of the materials (Figure 7b) [30]. S-PLA formation increases the thermal degradation temperature owing to the strong interactions between the L- and D-lactide chains, which significantly reduces molecular mobility and disturbs thermal degradation [106,107]. The bio-stereo-nanocomposites PLA–CNW materials s-PLA–CNW1 and s-PLA–CNW3 showed enhanced thermal degradation properties as compared with the original s-PLA, indicating that the CNW particles can act as a superior insulator and mass transport barrier during the thermal degradation process. The enhancement of these valuable properties represents a synergetic effect of the strong interaction among the molecules in s-PLA crystallites and well-distributed CNW particles in bio-stereo-nanocomposite PLA–CNW.

One of the important properties of stereocomplex materials related to processing materials is stereocomplex memory or melt stability. Stereocomplex memory is the ability of stereocomplex materials to reform stereocomplexes after they are melted. Because its unzipped fragment cannot re-zip to form s-PLA after melting, stereocomplex memory is the main limitation of linear s-PLA [14,106]. Some structural modifications were developed to solve this limitation [14,106,108]. Biela et al. reported that a star-shaped s-PLA with a minimum of 13 arms could maintain stereocomplex memory through parallel or anti-parallel orientations of hardlock-type interactions [106]. Star-shaped PLA with a smaller arm number (8 arms) and a three-dimensional core structure also shows excellent stereocomplex memory because a precise position and space distribution minimizes the movement of the lactide chains [108].

**Figure 8:**
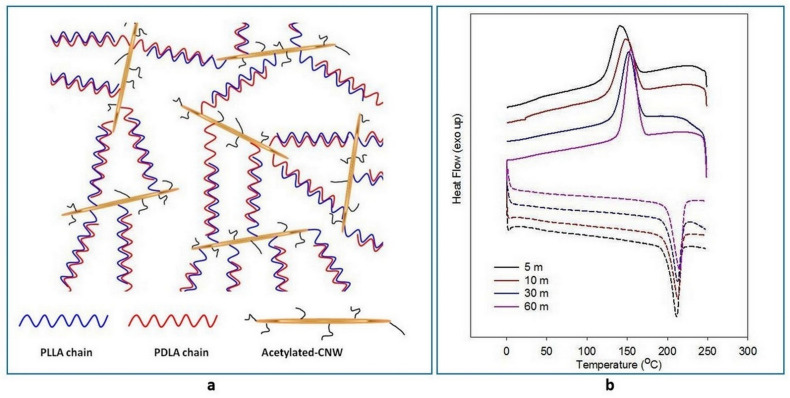
(**a**) Schematic structure of s-PLA–CNW via antiparallel re-assembles supported by graft-structure and well-distributed acetylated-CN; (**b**) DSC thermograms of bio-stereo-nanocomposites PLA–CNW at various processing times; cooling (solid-line) and second heating (dash-line). (Copyright and permission, [29]).

The bio-stereo-nanocomposites from CNW-grafted PLLA and PDLA show excellent stereocomplex memory, which is supported by the graft structure of supramolecules similar to the star-shaped structure and the presence of the CNW particles’ three-dimensional core structures. Figure 8a shows the mechanism of stereocomplex memory through anti-parallel interaction, as supported by the highly distributed CNW particles [29]. The stereocomplex memory was confirmed through the presence of a single peak during the cooling process and by performing a second scanning of DSC analysis (Figure 8b) [29]. The grafted structure of the PLA–CNW materials minimized the freedom of the PLLA and PDLA chains during the melting process. Consequently, the unzipped PLLA and PDLA chains could easily re-zip after melting. This is also supported by the well-distributed CNW particles as nucleating agents.

The combination of stereocomplex crystallization and nanocomposite approaches brings simultaneous improvements in the mechanical and thermal properties of PLA, including stereocomplex memory and an unusual stereocomplex crystallization process. The simultaneous property enhancement is attributed to the synergistic effect derived from the nucleating agent support of well-dispersed CNW particles and chain freedom of PLLA and PDLA. PLA chains can be modified using various polymer chains and structures; thus, the bio-stereo-nanocomposites PLA–CNW has numerous general and specific applications and found an important role in food packaging, automotive, engineering, and biomedical applications owing to their unique properties [109,110,111,112,113]. Controlling CNW miscibility in PLA matrix helps enhance the mechanical, optical, and thermal properties, which are the main requirements for food packaging applications [109,110,111]. As a bio-based material, PLA–CNW with its biodegradability and cytocompatibility fulfills the basic requirements for use in biomedical applications [112,113] and is a potential candidate for the development of eco-friendly materials in the future owing to its sustainability (from renewable resources), biodegradability, and property tune-ability. Highly functional nanomaterials can be generated from PLA and CNW, thereby expanding the advanced and highly specific applications, such as biomedical and tissue engineering applications.

## 5. Conclusions and Prospective

PLA and CNW materials are a promising material for future applications as they are derived from sustainable resources and degradability. PLA and CNW can be developed as advanced materials through nanocomposite and stereocomplex approaches. The combination of PLA and CNW attracted great interest because they are fully bio-based materials. The development of fully-bio-based materials utilizing PLA and CNW materials through nanocomposite and stereocomplexation approaches. PLA–CNW nanocomposites can be obtained through solution casting and melt blending processes. The critical point of development of the PLA–CNW nanocomposites is mainly associated with the inherent hydrophilicity of CNW particles. Unmodified CNWs improve the PLA properties with limited CNW dispersibility in the PLA matrix. Chemical modifications on the CNW surface to control the –OH groups improved the dispersion and compatibility of CNWs in the PLA matrix. Acetyl groups, esterification, and grafted-PLA chains on the CNW surface increase the interfacial interaction with the polymer matrix through hydrogen bonding. Well-distributed CNW particles in PLA–CNW nanocomposites can act as a nucleating agent to improve the PLA crystallinity, thereby affecting the thermal, mechanical, and barrier-related properties.

Bio-stereo-nanocomposite PLA–CNW is an advanced material developed using stereocomplexation and nanocomposite approaches. The presence of CNW particles in high-molecular-weight linear PLLA and PDLA homopolymer blends constrained by the molecular weight resulted in the formation of partial stereocomplex crystallites. Bio-stereo-nanocomposite PLA–CNW from CNW-grafted PLLA and PDLA shows unusual stereocomplex formation in a very short time and excellent stereocomplex memory after melting. These graft structures presumably provided a three-dimensional core structure and minimize the chain mobility of PDLA and PLLA. The stereocomplex crystallites and nucleating agents from CNW particles contribute to the simultaneous improvement of the properties of bio-stereo-nanocomposites PLA–CNW. The simultaneous enhancement of the properties opens the window to be applied in a wide range of industrial applications. Bio-based materials from PLA and CNW can be used in the development of future materials owing to their properties of biodegradability, sustainability, and property tunability. Moreover, PLA and CNW have the ability to be modified with specific functional structures. With its tacticity and functionality, PLA has a great chance for molecular modifications and stereocomplexation. The surface hydroxyl groups on the CNW surface can be controlled to adjust the hydrophobicity and surface functionality. These properties of PLA and CNW bring bio-based PLA–CNW promising for the development of future materials for specific and advanced applications.

## Figures and Tables

**Table 1:** Properties of PLA, PS, and PET.

Properties	PLLA	PS	PET
Density (kg m^−3^)	1.26	1.05	1.40
Tensile strength (MPa)	59	45	57
Elastic modulus (GPa)	3.8	3.2	2.8–4.1
Elongation at break (%)	4–7	3	300
Notched izod (J m^−1^)	26	21	59
Heat deflection (°C)	55	75	67

**Table 2:** Polymer matrix, surface modification, processing, and properties of PLA–CNW nanocomposites.

Polymer Matrix	CNW Modifications/Synthesis	Nanocomposites Processing	Properties Improvement	Ref. (Year)
PLA (*M*_w_ = 100,000 g/mol)	Acid hydrolysis from cotton fabric	Solution casting in chloroform/N,N’dimethylformamide	Thermal stability, tensile strength, and Young’s modulus	[22] (2021)
PLA (*M*_n_ = 130,000 g/mol)	Acid hydrolyzed from cellulose	Solution casting in chloroform	Water permeability and oxygen permeability	[26] (2010)
PLA	Acid hydrolyzed from flax cellulose	Solution process in N,N’dimethylformamide to form nanofibrous mat	Crystallinity and water absorption	[27] (2018)
PDLLA	Acid hydrolyzed from eucalyptus kraft wood pulp	Solution casting in dimethylformamide	Hydrolytic degradation, thermal stability	[81] (2011)
PLA	Acetylation CNW	Solution polymerization Bulk Polymerization Solution Blending	Thermal stability and Crystallinity	[18] (2012)
PLA (*M*_w_ = 52,000 g/mol)	Acetylation using acetic anhydride	Solution casting in chloroform	Tensile strength, thermal stability, dimensional stability, and dynamic mechanical properties	[25] (2014)
PLA (*M*_w_ = 180,000 g/mol)	Acetylation CNW	Solution casting in dichloromethane	Stress transfer between CNW and PLA matrix	[82] (2017)
PLA (*M*_n_ = 1.0 × 10^5^ g/mol)	Surface esterification by formic acid	Solution casting in chloroform	Barrier properties	[83] (2017)
PLA (*M*_w_ = 87,000 g/mol)	Grafted toluene diisocyanate	Solution casting in chloroform	Tensile strength	[84,85] (2016)
PLA (*M*_w_ = 100,000 g/mol)	Surface modification by triazine derivative	Hot compression process 170oC 40 MPa	Breaking strength, elongation, compatibility, and thermal properties	[23,86] (2017, 2018)
PLA (*M*_n_ = 98,000 g/mol; *M*_w_ = 199,590 g/mol)	Surface esterification by benzoic acid	Masterbatch followed by extrusion process	The Young’s modulus and ultimate tensile stress	[73] (2018)
PLA (*M*_n_ = 98,000 g/mol; *M*_w_ = 199,590 g/mol)	Surface esterification by valeric acid	Masterbatch followed by extrusion process	Thermal decomposition, mechanical properties, and crystallinity growth	[74] (2019)
PLA (*M*_w_ = 2.1 × 10^5^ g/mol)	Graft modification by 3-aminopropyltriethoxysilane	Solution casting in dichloromethane	Air permeability, light resistance, thermal stability, and mechanical properties	[87] (2020)
PLA (*M*_n_ = 150,000 g/mol)	Addition radical initiator with dicumyl peroxide	Reactive extrusion by Twin-screw extruder	Mechanical properties, crystallinity. processability, melt-strength, rheological behavior	[88,89] (2016, 2017)
PLLA (*M*_w_ = 100,000 g/mol)	Grafted lactic acid	Solution casting in chloroform	Tensile strength and Young’s modulus	[75] (2018)
PLA (*M*_n_ = 95,000 g/mol) PLA (*M*_n_ = 130,000 g/mol)	Grafting PLLA by ring-opening polymerization in toluene	Melt-blending in mini extruder	Compatibility, thermal behavior, and mechanical properties	[58] (2011)
PLA	Grafting PLLA by ring-opening polymerization in toluene	Twin-screw extruder	Thermal, mechanical, optical, and morphological properties	[84] (2016)
PLA	Grafting PLLA by ring-opening polymerization in dimethyl sulfoxide	Solution casting and co-extrusion	Barrier and dynamic mechanical properties	[90] (2016)
PLA (*M*_n_ = 110,000 g/mol)	Grafting PLLA by ring-opening polymerization in toluene	Melt spinning using twin-screw micro-compounder	Thermal stability, degree of crystallinity, and mechanical properties	[91] (2016)

Note: *M*_n_: number average molecular weight; *M*_w_: weight average molecular weight.

## Data Availability

Data is contained within the article.
